# Impact of COVID-19 on Mucosal Immunity and Antibody Responses in COVID Vaccinees

**DOI:** 10.3390/vaccines13090967

**Published:** 2025-09-12

**Authors:** Priya Kannian, Muruganantham Lillimary Eniya, Pasuvaraj Mahanathi, Arul Gracemary, Nagalingeswaran Kumarasamy, Stephen J. Challacombe

**Affiliations:** 1 VHS Laboratory Services, The Voluntary Health Services, Chennai 600113, India; enianand98@gmail.com (M.L.E.);; 2 Infectious Diseases Medical Centre, The Voluntary Health Services, Chennai 600113, India; kumarasamy@cartcrs.org; 3 Faculty of Dentistry, Oral & Craniofacial Sciences, King’s College London, London SE1 9RT, UK; stephen.challacombe@kcl.ac.uk

**Keywords:** SARS-CoV-2, anti-SARS-CoV-2 spike antibodies, Covishield vaccine, cytokines, secretory IgA, mucosal immunity

## Abstract

**Background and Objectives**: SARS-CoV-2 infection initiates at mucosal surfaces, and mucosal immunity may influence the nature and severity of infection. Little is known about the induction of mucosal immunity by vaccination in COVID-19 convalescents. **Methods**: Sera from 205 healthcare workers were collected one month after the first Covishield vaccination and 1/3/6 months after the second vaccination, while paired sera and stimulated whole-mouth fluid (SWMF) was collected 1/3/6 months after the third vaccination (N = 10) and at 0/30/90 days after a COVID-19 episode (N = 8). Anti-SARS-CoV-2 spike antibody detection by ECLIA/ELISA and cytokine detection by ELISA/CBA were performed. **Results**: One month post-second vaccination, serum antibodies had increased significantly (6-fold) in the COVID-19-naïve group (CNG) but declined (1.5-fold) in the previously COVID-19-exposed group (CEG), who already had high antibody titres. The serum regulatory cytokine IL-10 levels were higher after three antigen exposures (*p* = 0.0002). New infections (breakthrough infections—BTIs) or reinfections (RIs) with asymptomatic/mild disease occurred in 44% of the CNG and 27% of the CEG (*p* < 0.01). The mucosal cytokine IL-17 levels were significantly higher in the CEG. Salivary IgG/IgA and secretory IgA antibodies were detectable both after vaccination and COVID-19. Innate cytokines (MIG, MCP-1, IL-8, IL-1β) were higher and sustained in SWMF in contrast to serum. **Conclusions**: Two vaccinations in the CNG resulted in an antibody boost, but the second vaccination in the CEG induced antibody anergy. Serum/mucosal antibodies declined by six months after vaccination, but the rapid increase at subsequent exposures were indicative of a good T cell/B cell memory response to SARS-CoV-2. A higher percentage of BTI among the CNG than RI among the CEG may indicate better protection due to higher antibody responses in the latter group.

## 1. Introduction

Vaccines confer protection by inducing certain host effector mechanisms, including antibodies, cytokines, and chemokines produced by B cells, CD8^+^ T cells, and CD4^+^ T cells. The CD4^+^ helper T cells (Th cells) mediate protection through cytokines and contribute to the generation and maintenance of B cells and CD8^+^ T cells. CD4^+^ effector T cells that play a major role in vaccine-induced protection include the follicular helper T cells (Tfh cells), Th17 cells, and regulatory T cells (Tregs). Tfh cells are primarily positioned in the local lymph nodes where they mediate B cell activation, differentiation, somatic hypermutation, and class switching. They directly control the antibody responses and mediate adjuvanticity. Th17 cells promote a local pro-inflammatory response and contribute largely to mucosal immunity. These effector T cells are controlled by the Tregs. The cytokines (IL-17, IL-21, IL-10, IL-7, IL-15) produced by these effector and regulatory T cells contribute to the effectiveness of a vaccine [1,2,3,4].

The predominantly administered COVID-19 vaccine in India is the Covishield vaccine, which is identical to Astra Zeneca’s ChAdOx1 and is manufactured under a licence. It is a chimpanzee adenoviral vector vaccine carrying the genetic material coding for the SARS-CoV-2 spike protein. Studies from India on vaccine efficacy have reported that the Covishield vaccine reduces the incidence rate of infection, transmission rate, mortality rate, and rate of hospitalization compared with unvaccinated controls [5,6,7,8].

Although these large cohort studies have shown the effectiveness of this vaccine, there is little knowledge about the T cell responses elicited. Robust T effector and memory cell responses are important for the activation of B cells and maintenance of memory B cells. Studies have demonstrated differential humoral and in vitro cell-mediated immune responses in COVID-19-naïve group (CNG) compared with COVID-19-exposed group (CEG) after two vaccinations with BNT162b2 and after one vaccination with Ad25.CoV2.S [9,10,11,12,13,14,15]. Since these studies were performed in vitro upon the stimulation of lymphocytes with specific peptides, these findings do not necessarily correlate with an in vivo reduction in antigen burden. In addition, there is very little data on the induction of mucosal immunity by the Covishield vaccination. Mucosal immunity represents both innate and adaptive immunity at all mucosal surfaces and can differ substantially from systemic immunity in the local production of antibodies, lymphocyte distribution, and cytokines. It may be the primary defence against pathogens.

Therefore, in this study, we proposed to determine the antibody responses and the pro-inflammatory and memory T cell responses in Covishield vaccinees in the serum and mucosal secretion from stimulated whole-mouth fluid (SWMF) samples up to six months after a third vaccination. We also elucidated the mucosal immunity in a sub-group who developed COVID-19 after vaccination. We compared B cell and T cell responses to Covishield up to six months after two vaccinations in CEG and CNG with those in people who developed COVID-19 after vaccination. The innate and adaptive immunity cytokines in paired serum and SWMF samples were also determined one and 3–6 months after the third vaccination.

## 2. Materials and Methods

### 2.1. Patients and Samples

The study was approved by the VHS-Institutional Ethics Committee (proposal no. VHS-IEC/72-2020 and VHS-IEC/75-2021). Healthcare workers (HCWs; n = 220) from three different hospitals in Chennai, India, were recruited one month after the first vaccination of Covishield (identical to ChAdOx1 and manufactured in India) after obtaining written informed consent between January 2021 and October 2022. The SARS-CoV-2 strains prevalent in India during this time were Delta (B.1.617.2) and Omicron (B.1.1.529). Blood (by venipuncture) was collected at recruitment. Demographic details, past COVID-19 exposure, and vaccination dates were collected. The second and third vaccination time points were compliant with Indian government vaccination policies (initially a one-month gap, then three months, then 6–9 months based on vaccine availability). After the second vaccination, blood samples were collected at one month, three months, and six months. After the third vaccination, concomitant blood and stimulated whole-mouth fluid (SWMF) samples were collected at one month, three months, and six months. In a small subset of eight HCWs who developed mild COVID-19, additional blood and SWMF samples were collected at the onset of disease, as well as one month and three months after the onset of disease. Of these eight HCWs, 2 (25%) developed COVID-19 for the first time post-vaccination, 4 (50%) developed a second COVID-19 episode, and 2 (25%) developed COVID-19 for the third time.

For SWMF samples, the participants were requested to chew a sterile inert paraffin wax for five minutes and drool the collected saliva during that period into a sterile wide-mouthed container. SWMF samples were refrigerated and processed within six hours as previously described [16]. Sera and SWMF supernatant samples were separated immediately by centrifugation (serum: 3500 rpm for 25 min; SWMF: 1500 rpm for 10 min) and stored at −80 °C until further use. HCWs lost to follow-up beyond one month after the first vaccination (n = 15) were excluded from the study. Additionally, SWMF samples were collected from 11 unvaccinated COVID-19-naïve healthy controls (selection criteria included having had no known clinical symptoms of COVID-19 and being negative for anti-SARS-CoV-2 nucleoprotein antibodies).

### 2.2. Anti-SARS-CoV-2 Spike and Nucleoprotein Antibodies

Anti-SARS-CoV-2 spike and/or nucleoprotein antibodies (non-isotype specific but mainly IgG as per the manufacturer) were detected by an electrochemiluminescence assay (ECLIA) against recombinant SARS-CoV-2 proteins using a Cobas e411 automated analyzer (Roche, Penzberg, Germany). Values < 1 from the cut-off index (COI; lower limit of detection was 0.4 COI) were considered negative for the anti-nucleoprotein antibody kit. Values < 1 U/mL (lower limit of detection was 0.4 U/mL) were considered negative for the anti-spike Ig antibody assay. Samples with values > 250 U/mL (analytical measurement range is 0.4–250 U/mL) were serially diluted to 1:400 with Dulbecco’s phosphate-buffered saline (PBS; HiMedia, Mumbai, India), and the final antibody concentration was determined.

### 2.3. SARS-CoV-2 Spike-Specific IgG ELISA

Sera (dilution 1:2 to 1:32,000) and SWMF (dilution 1:2 to 1:400) samples were tested for anti-SARS-CoV-2 spike IgG by the commercially available Wuhan-Hu-1 Human SARS-CoV-2 Spike (trimer) IgG ELISA Kit (Invitrogen, Waltham, MA, USA) according to the manufacturer’s instructions. Samples were diluted until the OD values fell onto the linear part of the standard curve.

### 2.4. SARS-CoV-2 Spike-Specific Total IgA ELISA

Sera (dilution 1:2000 to 1:16,000) and SWMF (dilution 1:20 to 1:160) samples were tested for anti-SARS-CoV-2 spike RBD total IgA using an in-house ELISA. High-binding microtiter plates (R&D Systems, Minneapolis, MN, USA) were coated with 0.5 μg/mL recombinant SARS-CoV-2 spike RBD His tag antigen (R&D Systems, Minneapolis, MN, USA). Plates were incubated overnight at room temperature (RT). After washing with 1× PBS (HiMedia, Mumbai, India) containing 0.05% Tween 20 (PBST; Merck, Rahway, NJ, USA), the pates were blocked for 1 h with 1% milk in PBS. Four serial dilutions of the samples were added and incubated for 2 h at RT. After washing with PBST, goat anti-human IgA (α chain) antibody–biotin conjugate (0.2 μg/mL GOXHU IgA Bio, Invitrogen, USA) was added and incubated for 2 h at RT. After washing with PBST, Streptavidin-HRP-A (R&D Systems, Minneapolis, MN, USA) was added for 20 min at RT. The plates were washed with PBST, and 3,3′,5,5′-Tetramethylbenzidine (TMB; R&D Systems, Minneapolis, MN, USA) was added for 20 min at RT; the reaction was stopped with 2N sulphuric acid (R&D Systems, Minneapolis, MN, USA), and the OD values were measured at a wavelength of 450 nm and a reference wavelength of 550 nm.

Four highly positive sera were chosen and pooled to generate the standard curve. Seven serial 2-fold dilutions were made in duplicate for the standard curve from 1:1000 of the pooled sera. The highest concentration of the standard curve was assigned an arbitrary value of 1000 U/mL. Baseline cut-off OD values of 0.2 for sera and 0.1 for SWMF samples were established using CRP-negative serum samples from healthy controls and SARS-CoV-2 RT-PCR-negative SWMF samples, respectively. An average of the replicates for each of the samples that were within the linear part of the standard curve was used as the final value in U/mL.

### 2.5. SARS-CoV-2 Spike-Specific Secretory IgA (sIgA) ELISA

SWMF (dilution 1:2 to 1:16) samples were tested for anti-SARS-CoV-2 spike RBD sIgA using an in-house ELISA. High-binding microtiter plates (R&D Systems, Minneapolis, MN, USA) were coated with 0.5 μg/mL recombinant SARS-CoV-2 spike RBD His tag antigen (R&D Systems, USA) overnight at RT. After washing and blocking as stated above, four serial dilutions for each of the samples were added and incubated for 2 h at RT. The plates were washed, and mouse anti-human secretory IgA monoclonal antibody (2.5 μg/mL; MC29-12, Invitrogen, Waltham, MA, USA) was added and incubated for 2 h at RT. After washing, goat anti-mouse IgG (H+L) secondary antibody–biotin conjugate (0.065 μg/mL, Invitrogen, Waltham, MA, USA) was added and incubated for 2 h at RT, and the plates were then treated as described above.

Four highly positive SWMF samples were chosen and pooled to generate the standard curve. Seven serial 2-fold dilutions were made in duplicate for the standard curve from 1:2 of the pooled SWMF sample. The highest concentration of the standard curve was assigned an arbitrary value of 500 U/mL. A baseline cut-off OD value of 0.05 was established using the same SARS-CoV-2 RT-PCR-negative SWMF samples used for the total IgA assay above. An average of the replicates for each of the samples that were within the linear part of the standard curve was used as the final value in U/mL.

The antibody levels (IgG, IgA, sIgA) in the SWMF samples were normalized over the salivary flow rate of the corresponding samples by dividing the antibody value by the salivary flow rate and values expressed as U/mL/min.

### 2.6. Cytokine Assays

IL-17, IL-21, and IL-10 levels were tested at one month after each vaccination, while the levels of memory cytokines IL-7 and IL-15 were tested 3–6 months after the second vaccination. IL-21, IL-7, IL-10, and IL-15 were determined by an enzyme-linked immunosorbent assay (ELISA), according to the manufacturer’s instructions (R&D Systems, Minneapolis, MN, USA). Cytokine bead arrays (CBAs) were performed on the paired serum and SWMF samples collected from 18 HCWs after three antigen challenges (either by vaccination and/or natural infection) using flow cytometry and the BD CBA flex beads for IL-2, IL-4, IL-12p70, IFN-γ, MIG, MCP-1, IL-8, TNF-α, IL-6, IL-1β, and IL-17 as per the manufacturer’s instructions (BD FACS Lyric, BD Biosciences, San Jose, CA, USA). For SWMF samples, the total protein content was determined by the pyrogallol red molybdate method (Dimension Xpand, Siemens Healthcare, Malvern, PA, USA) to normalize the cytokines and chemokines in the SWMF samples. The cytokine levels (pg/mL) in the SWMF samples were divided by the total protein concentration (mg/mL) in the SWMF and expressed as pg/mg of the total protein.

### 2.7. Statistical Analysis

The mean, median, standard deviation, and quartile values were calculated using Microsoft Excel and Graphpad Prism 8.0. The statistical significance between the groups was calculated using Graphpad Prism 8.0 and the free online calculators from Social Science Statistics or Vassar Stats. Gender and age comparisons were performed using chi-squared tests and comparisons of independent means and paired means by the *t*-test or ANOVA test. Comparisons of the medians in the box-and-whisker plots were performed using the Mann–Whitney U test.

## 3. Results

Of the 205 cases analyzed in this study, 111 (54%) were in the CNG and 94 (46%) were in the CEG prior to vaccination. Demographic details of the 205 cases are shown in Table 1. The number of females recruited in the study was almost three times that of the males. There were no significant gender or age differences between the CNG and CEG. However, a higher percentage of breakthrough infections (BTIs; *p* = 0.007) or re-infections (RIs; *p* = 0.008) was found in males compared to the females (Table 1).

### 3.1. Antibody Anergy in Previously COVID-19 Exposed Vaccinees but Not in Previously Vaccinated COVID-19 Patients

**Figure 1:**
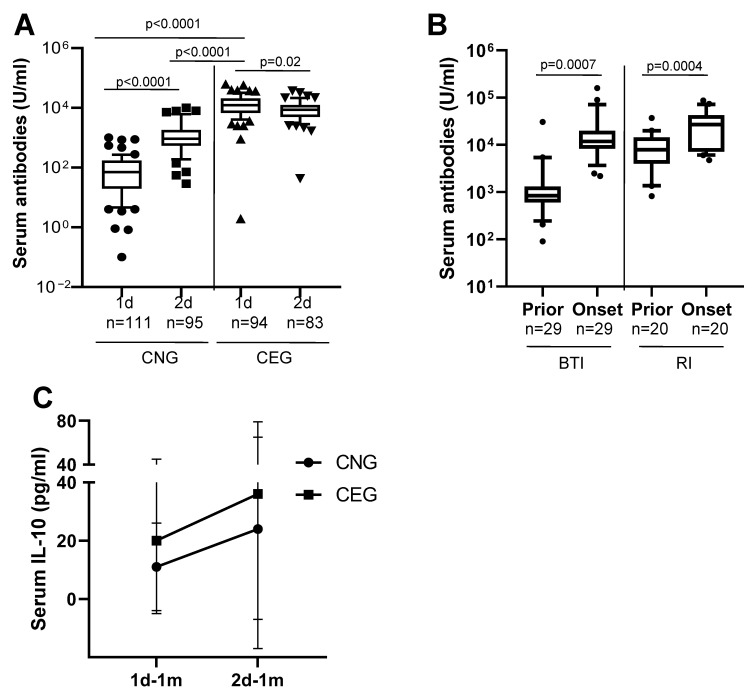
Serum anti-SARS-CoV-2 spike Ig antibodies and IL-10 levels in COVID-19-naïve (CNG) and COVID-19-exposed (CEG) groups. n is the number of samples in each group. (**A**) Antibodies at one month after the first and second vaccinations. The y-axis denotes the logarithmic concentration of the antibodies (U/mL). The box denotes 10th–90th percentile values of each group. Symbols denote the minimum and maximum values. The *p* values are determined by the Mann–Whitney U test. (**B**) Serum anti-SARS-CoV-2 spike Ig antibody responses prior to and at onset of breakthrough infection (BTI) and re-infection (RI). The y-axis denotes the logarithmic concentration of the antibodies (U/mL). The box denotes 10th–90th percentile values of each group. Symbols denote the minimum and maximum values. The *p* values are determined by the paired *t*-test. (**C**) Levels of IL-10 in the CNG and CEG. Black circles—CNG; black squares—CEG. The y-axis denotes the concentration of serum IL-10 (pg/mL). The error bars indicate the mean ± SD for each group. The *p* values are determined by the paired *t*-test.

Among the CNG, 78/111 (70%) developed serum anti-SARS-CoV-2 spike Ig antibodies detectable at one month after their first vaccination and 104/111 (94%) at one month after the second vaccination. The median serum antibody response at one month after the first vaccination in the CEG (median: 10,152 U/mL) was significantly greater than in the CNG (median: 84 U/mL; *p* < 0.0001; Figure 1A; Table 2). The serum antibody response in the CNG at one month after the second vaccination (median: 856 U/mL) was still significantly lower than the antibody response in the CEG at one month after the first vaccination (median: 10,152 U/mL; *p* < 0.0001; Figure 1A; Table 2). The serum antibody response in the CNG showed a statistically significant 6-fold increase at one month after the second vaccination (Figure 1A; Table 2) compared with the first vaccination (*p* < 0.0001). In contrast, the median antibody level in the CEG showed a statistically significant decline at one month after the second vaccination (median: 6986 U/mL; Figure 1A; Table 2) compared with the first vaccination (*p* = 0.02). These findings suggest that the second vaccine dose boosts the antibody response in the CNG, while it causes antibody anergy in the CEG, and a natural SARS-CoV-2 infection results in higher antibody response than vaccination.

Among the CNG, 49/111 (44%) had developed BTIs that were either mildly symptomatic (RT-PCR or RAT positive using nasopharyngeal swabs) or had asymptomatic infection (had become anti-SARS-CoV-2 nucleoprotein antibody-positive at six months in the absence of symptoms). The disease severity was determined as per the NIH COVID-19 severity scores [17]. Among the vaccinated CEG, 25/94 (27%) developed RIs that were either mildly symptomatic (RT-PCR or RAT positive) or asymptomatic. The number of BTIs in the CNG were significantly greater than the RIs in the CEG (*p* = 0.01; chi-squared test with Yates correction for continuity). Longitudinal serum samples 3–6 months prior to the onset and after the onset of BTIs or RIs were available for an analysis of anti-SARS-CoV-2 spike-specific antibody levels in 29 CNG and 20 CEG individuals, respectively. The median antibody level in the BTI group prior to onset was 840 U/mL and after onset was 11,800 U/mL (*p* = 0.0007). In the RI group, the median antibody level prior to onset was 7924 U/mL and after onset was 26,920 U/mL (*p* = 0.0004; Figure 1B). This suggests that a SARS-CoV-2 infection in COVID-19-vaccinated individuals results in an increase in specific antibody response.

IL-10 is a regulatory cytokine that decreases T cell mediated pro-inflammatory response. Figure 1C shows that the IL-10 levels increase with every antigen challenge (infection or injection). The mean serum IL-10 level at one month after the first vaccination (1d-1m; Figure 1C) was greater in the CEG (mean: 20 pg/mL) compared with the CNG (mean: 11 pg/mL). At one month after the second vaccination (2d-1m), the IL-10 levels in the CNG (mean: 24 pg/mL) had increased 2-fold from 1d to 1m and were now similar to those of the CEG at the 1d-1m time point. In the CEG, the IL-10 levels at 2d-1m (mean: 36 pg/mL) increased markedly from 1d to 1m (*p* = 0.04; Anova test). Additionally, the IL-10 levels in the CEG at 2d-1m after three antigen challenges was statistically higher than the CNG 1d-1m (*p* = 0.0002; ANOVA test). Thus, an incrementing T cell regulatory response is evident with every antigen challenge.

### 3.2. Antibody Memory to SARS-CoV-2 and Protection

**Figure 2:**
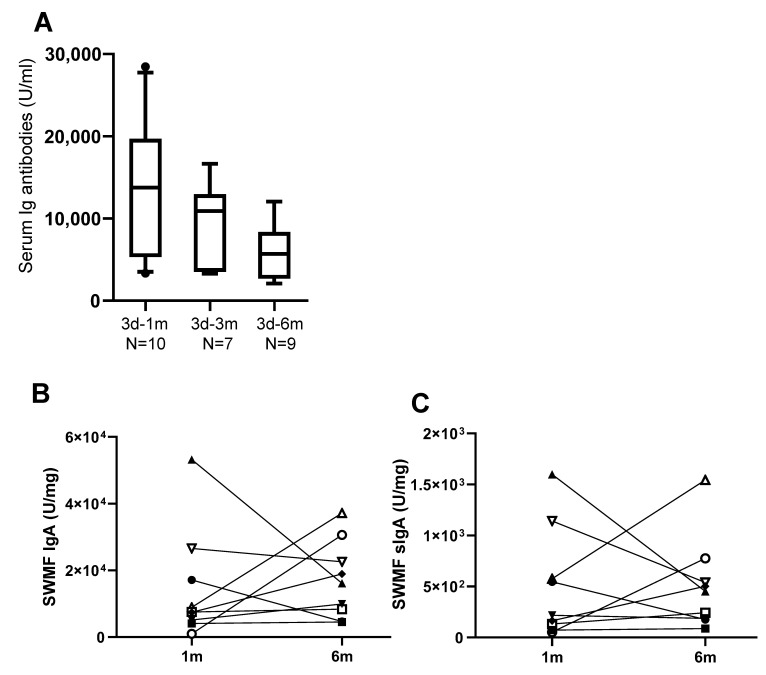
Anti-SARS-CoV-2 spike antibody elicitation in SWMF after COVID-19 vaccination. N = number of samples in each group; d denotes vaccination dose; and m denotes months. (**A**) Anti-SARS-CoV-2 spike Ig antibodies after the third vaccination in serum. The y-axis denotes the concentration of the antibodies (U/mL). The box denotes 10th–90th percentile values of each group. Symbols denote the minimum and maximum values. (**B**) Anti-SARS-CoV-2 spike IgA antibodies in SWMF samples. Each HCW is denoted by a different symbol. (**C**) Anti-SARS-CoV-2 spike sIgA antibodies in SWMF samples. Each HCW is denoted by a different symbol.

A subset of 10/205 HCWs who were vaccinated for the third time were tested for serum and mucosal anti-SARS-CoV-2 spike antibodies (Figure 2). The median serum Ig antibody titre at one month after the third vaccination (3d-1m; median: 13,760 U/mL) was similar to the Ig antibody levels in the CEG at 1d-1m (median: 10,152 U/mL), suggesting a boost in antibody responses. Serum antibodies declined by 3–6 months (Figure 2A) after the third vaccination. The trends of the rise and decline of the serum antibodies after three vaccinations were similar to that after two vaccinations. Taken together, the data suggests that there is an antibody memory in the host immune system that gets activated upon subsequent exposures, and the antibody levels are short-lived.

### 3.3. Mucosal Antibodies Elicited by Vaccination/Natural Infection

We next measured the mucosal IgA (monomer + dimer) and the secretory IgA (sIgA; dimer) antibodies against SARS-CoV-2 spike protein in SWMF samples of the 10 HCWs who took the third vaccination at one month and six months. IgA (Figure 2B) and sIgA antibodies (Figure 2C) were detectable at both the time points tested and declined in most of the HCWs by six months.

**Figure 3:**
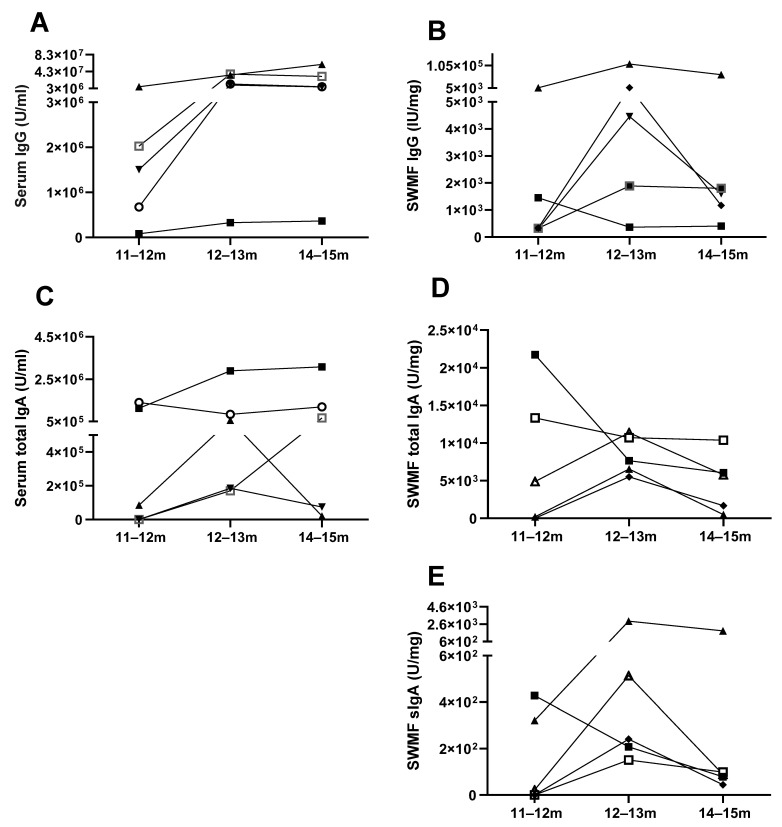
Longitudinal trends of IgG, IgA, and sIgA antibodies in serum and SWMF of five vaccinated HCWs after an episode of COVID-19. Each HCW is denoted by a different symbol. m denotes months. (**A**) Serum IgG antibodies. (**B**) SWMF IgG antibodies. (**C**) Serum IgA antibodies. (**D**) SWMF IgA antibodies. (**E**) SWMF sIgA antibodies.

Additionally, we followed 5/205 vaccinated HCWs through an episode of COVID-19 up to three months from disease onset. Anti-SARS-CoV-2 spike IgG, IgA, and sIgA antibodies were tested in serum and SWMF at onset, one month, and three months, which was 11–12 months, 12–13 months, and 14–15 months, correspondingly, from the time of their first COVID vaccination. Both serum IgG (Figure 3A) and serum IgA (Figure 3C) antibodies showed a similar trend of increase in levels by 12–13 months and then a decline by 14–15 months in the majority (Figure 3). SWMF IgG and IgA antibody levels are shown in Figure 3B and Figure 3D, respectively. The majority showed a similar pattern of an increase in SWMF sIgA antibodies at one month and a decrease at three months (Figure 3E).

**Figure 4:**
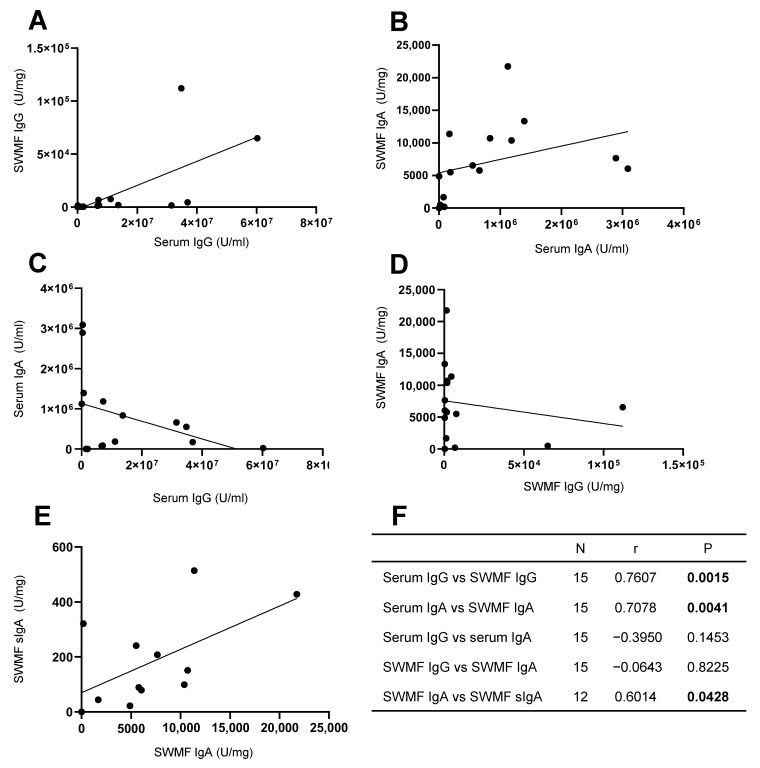
Correlation of anti-SARS-CoV-2 spike IgG, IgA, and sIgA antibodies in concomitant serum and SWMF samples. (**A**) Serum IgG vs. SWMF IgG. (**B**) Serum IgA vs. SWMF IgA. (**C**) Serum IgG vs. Serum IgA. (**D**) SWMF IgG vs. SWMF IgA. (**E**) SWMF IgA vs. SWMF sIgA. (**F**) Table inset shows the Spearman rank correlation values. N is the number of samples; r is the correlation co-efficient, and *p* is the *p*-value.

We analyzed the relationship between the anti-SARS-CoV-2 spike IgG, IgA, and sIgA antibodies in serum and SWMF samples in 15 HCWs post vaccination with or without COVID-19 exposure (Figure 4). Statistically significant correlations were seen between the serum and SWMF IgG antibodies (Figure 4A) and IgA (Figure 4B) antibodies. The Spearman rank correlation and *p* values are given in Figure 4F as a table inset. There were negative correlations between IgG (Figure 4C) and IgA (Figure 4D) antibodies in both the serum and SWMF, but these were not statistically significant. IgA and sIgA antibodies in SWMF showed a statistically significant direct correlation (Figure 4E). Thus, the passage of serum IgG and IgA antibodies into the oral cavity, as well as local sIgA antibody production, was evidenced.

### 3.4. Cytokines Indicate Sustained Innate Mucosal Immunity

**Figure 5:**
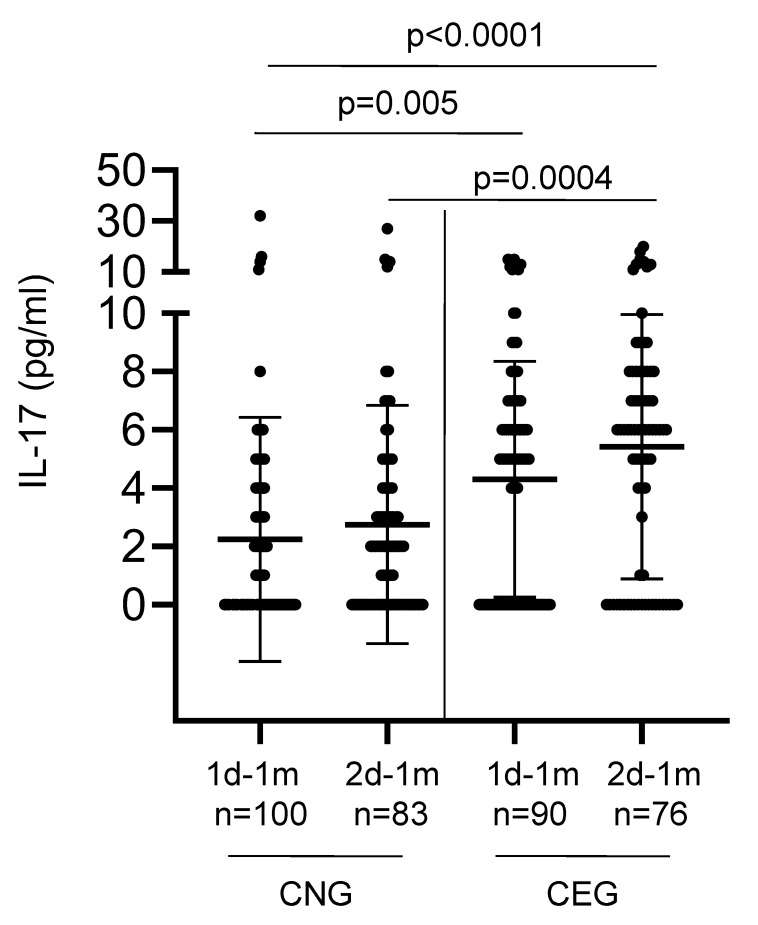
Levels of serum IL-17 detected in the COVID-19-naïve (CNG) and COVID-19-exposed (CEG) groups. The x-axis denotes the time points—1d-1m: one month after the first vaccination; 2d-1m: one month after the second vaccination. n denotes the number of samples. Bars show the mean ± SD for each group. The y-axis denotes the serum concentration of IL-17 in pg/mL. *p* values using ANOVA test for independent means.

We additionally determined the cytokines (IL-17, IL-21, IL-7, and IL-15) that are known to be secreted upon the activation of CD4^+^ helper T cells that are generally involved in the activation and maintenance of B cells. Low levels of serum IL-17 were detected in 106/190 (56%) vaccinees tested at one month after the first vaccination (Figure 5). The CEG elicited significantly higher levels of IL-17 compared with the CNG, both after the first (*p* = 0.005; ANOVA test) and second (*p* = 0.0004; ANOVA test) vaccinations. This increase in IL-17 (a key player in mucosal immunity) levels with every antigen challenge (either from the vaccine or natural infection) suggests a potential role for trained mucosal immunity.

Low levels of serum IL-21 were detected in 104/190 (55%) vaccinees at one month after the first (1d-1m) and second (2d-1m) vaccinations, with no significant difference between the CNG and CEG (Table 2). The mean IL-7 and IL-15 levels were also only marginally elevated in the vaccinees irrespective of the COVID-19 exposure before or after the vaccination (Table 2).

**Figure 6:**
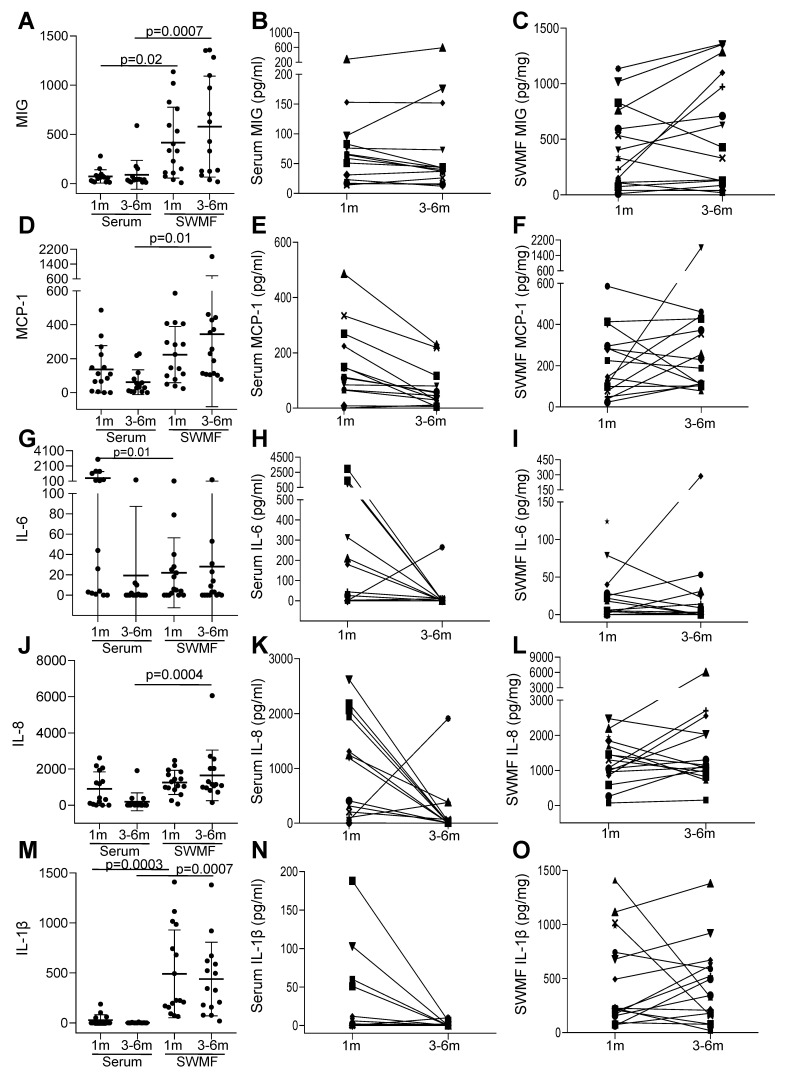
Comparison of cytokines and chemokines in serum and SWMF samples in 18 HCWs at one month (1 m) and 3–6 months (3–6 m) after the three antigen challenges. The y-axis denotes the concentration of the cytokines/chemokines in serum (pg/mL) and SWMF samples (pg/mg of total protein). The first column of graphs for each cytokine represents the mean and SD values along with the *p* values calculated by ANOVA at one month (1 m) and 3–6 months (3–6 m) in serum and SWMF samples. The second and third columns represent the longitudinal trends of the cytokines between 1 m and 3–6 m in serum and SWMF samples, respectively. Each symbol represents one participant. Monokine in response to interferon gamma (MIG (**A**–**C**)); monocyte chemoattractant protein-1 (MCP-1 (**D**–**F**)); interleukin-6 (IL-6 (**G**–**I**)); interleukin-8 (IL-8 (**J**–**L**)); interleukin-1β (IL-1β (**M**–**O**)).

Lastly, we analyzed the levels of selected innate and adaptive immunity mediators in the paired serum and SWMF samples from 18 HCWs after the three antigen challenges (vaccinations and/or natural infections). IL-2, IL-4, IL-12p70, IL-17, IFN-γ, and TNF-α were not detected in serum and SWMF samples at one and 3–6 months post-last vaccination. Cytokines and chemokines primarily secreted by monocytes and macrophages—MIG, MCP-1, IL-6, IL-8, and IL-1β were detected in both serum and SWMF samples (Figure 6). The mean levels of these cytokine/chemokine were significantly higher in SWMF than serum for MIG (1 m: serum-74 pg/mL; SWMF-417 pg/mg; *p* = 0.02; 3–6 m: serum-90 pg/mL; SWMF-580 pg/mg; *p* = 0.0007; Figure 6A), MCP-1 (1 m: serum-137 pg/mL; SWMF-224 pg/mg; 3–6 m: serum-61 pg/mL; SWMF-344 pg/mg; *p* = 0.01; Figure 6D), IL-8 (1 m: serum-910 pg/mL; SWMF-1262 pg/mg; 3–6 m: serum-189 pg/mL; SWMF-1653 pg/mg; *p* = 0.0004; Figure 6J), and IL-1β (1 m: serum-28 pg/mL; SWMF-491 pg/mg; *p* = 0.0003; 3–6 m: serum-2 pg/mL; SWMF-439 pg/mg; *p* = 0.0007; Figure 6M). In contrast, IL-6 levels were significantly higher in serum than SWMF at one month (1 m: serum-510 pg/mL; SWMF-22 pg/mg; *p* = 0.01; 3–6 m: serum-19 pg/mL; SWMF-28 pg/mg; Figure 6G), but by 3–6 months, the serum levels had declined significantly.

Additionally, a sustained trend in the SWMF samples between the first month and 3–6 months was found (Figure 6). In contrast to serum where levels of MCP, IL-6, IL-8, and IL-1β declined, those in SWMF were all maintained. IL-10 and IL-21 levels were detectable, but values were low. Taken together, this preliminary study on a subset of 18 HCWs suggests an innate immune response involvement, which lasts longer in the oral mucosa than the periphery.

## 4. Discussion

COVID-19 has provided key opportunities for understanding the immune responses to the disease with and without the context of vaccination. In this study, we were able to analyze the antibody responses in both serum and mucosal secretions and relate them to the subsequent susceptibility to SARS-CoV-2 infections (Figure 1B and Figure 3). The findings suggested that antibody levels in both fluids were directly related to protection from the severity of SARS-CoV-2 infection; furthermore, longer lived mucosal cytokine responses may also contribute to protection.

This study has found that two vaccinations in the CNG result in a systemic antibody boost and that a second vaccination in the CEG induces systemic antibody anergy, as demonstrated by a significant decline in the serum antibody levels after the second vaccination and increasing IL-10 levels. A similar anergic effect was shown in a study by Lozano-Ojalvo et al., where the antibody responses in the previously infected vaccinees plateaued after the first vaccination [9]. Thus, an antigen boost at the time of higher circulating antibodies causes antibody anergy. If the degree of protection is directly related to antibody titres, then the determination of circulating antibody levels before a vaccine boost may be beneficial [18]. However, this antibody anergy was not evident when natural infection occurred after vaccination. During a natural infection acquired post-vaccination, a range of functionally active antibodies against the invading virus are produced, whereas vaccinations activate only targeted B cell repertoires. This could potentially explain antibody anergy during vaccination post-natural infection but not vice versa.

Rapidly declining serum antibodies with minimal T cell cytokines raises concerns over their durability in subsequent virus exposures. The nature and extent of the antibodies induced by the Covishield vaccine in our study are similar to other studies with the Astra Zeneca vaccine, as well as other mRNA vaccines [9,19,20,21]. The antibody responses elicited by a natural infection was significantly higher than those elicited by the vaccination, as shown in the CEG, BTIs, and RIs. The serum antibody response elicited in the CNG after two vaccinations indicates a prime-boost response. This booster response was evidenced after each subsequent vaccination. The antibodies also declined proportionately by 3–6 months after either vaccination and/or natural infection. Our findings corroborate the finding of poor germinal centre formation shown at the tissue level in autopsy samples of acute COVID-19 patients [22]. Germinal centres are formed in the local inflammatory milieu primarily by Tfh cells where the B cells get activated. Kaneko et al. have shown that the accumulation of non-germinal centre-activated B cells in acute SARS-CoV-2 infection could be responsible for lower quality and less durable antibody responses [22]. However, studies have shown that T cells and B cells from vaccine-primed individuals can be induced in vitro by SARS-CoV-2 spike peptides [9,10,23], suggesting the presence of memory T cell and B cell pools. Our findings of a robust increase in antibody levels at the onset of COVID-19 in vaccinees reiterate the presence of a T cell/B cell memory pool; however, the circulating antibodies are short-lived in the host, raising concerns over a number of host factors that govern robust humoral immunity. These include inducing long-lasting antibody titres that are durable and highly avid, isotype changes, and the protective effect of serum antibody titres at mucosal surfaces. B cells are activated to elicit a humoral response both by the antigen itself as well as by the antigen-specific T cells. Only a T cell-dependent B cell response can provide long-lasting immunity with a robust memory pool [1].

Natural SARS-CoV-2 infection elicited a higher serum antibody response post-vaccination. Similarly, vaccination post-SARS-CoV-2 infection boosted significantly higher antibody levels. Recent studies have shown that the neutralizing activity of serum antibodies is higher in vaccinees exposed to natural infection and that these antibodies can also neutralize other human coronaviruses [24,25,26,27,28]. Thus, the breadth and magnitude of the serum antibody responses after a natural infection are higher than that of vaccination. The higher antibody responses could be attributed to exposure to a larger repertoire of antigenic epitopes and to a longer stimulus due to potential biological amplification of the virus in the host. The wider breadth of the antibody responses could be attributed to immunological imprinting due to immunological memory and antigenic seniority, as shown in other studies [29,30,31,32].

Higher class switching to IgA antibodies, the presence of IgA and sIgA antibodies in the oral mucosa, and sustained innate cytokines emanating from the oral mucosa may result in earlier virus clearance and influence quicker remission [33,34,35,36]. A rise and decline antibody pattern in concomitant serum and SWMF samples in eight vaccinees after the third vaccine dose suggests that there is passage of the antibodies into the oral cavity, probably through the crevicular spaces in the teeth pockets, which would confer local protection during subsequent virus exposures. Subsequent to either infection or vaccination, there was a minimal elicitation of IL-17, an important cytokine responsible for mucosal immunity. Even though the IL-17 levels were quite minimal, there was a statistically higher response in the CEG compared to the CNG. Overall mucosal immunity may be very relevant in protection against infection, and the presence of anti-SARS-CoV-2 antibodies (especially sIgA) in the SWMF samples found in this study could contribute to such protection at mucosal surfaces. However, a small sample size poses limitations for firm conclusions. Utilizing the SWMF samples to understand the role of mucosal immunity against SARS-CoV-2 may be productive [8].

## 5. Conclusions

Two vaccinations in the CNG result in a systemic antibody boost, but a second vaccination in the CEG induces systemic antibody anergy, while a BTI or RI in vaccinees boosts antibody levels presumably due to the immunogenic effects of an active virus infection. Asymptomatic/mild COVID-19 in BTI/RI cases are suggestive of a protective role for the specific antibodies. The specific antibody responses are short-lived, probably due to the minimal elicitation of T cell/B cell cytokine responses. However, the marked antibody increase after every exposure (vaccination/COVID-19) suggests an underlying robust T cell/B cell memory pool.

Increasing serum IL-17 levels with subsequent exposures and the presence of IgA/sIgA antibodies specific to the spike protein, as well as sustained innate immunity cytokines in the oral cavity, indicate a robust mucosal immune response. The observation that complete protection was not achieved in some individuals could be attributed to viral factors such as mutations of the structural proteins or host factors, including changes in the titre, avidity or isotype of the antibodies, or that antibodies are not present in sufficient amounts at the mucosal sites of infection. Further in-depth explorations of the mucosal innate immune response at various stages of the disease may be informative.

## Figures and Tables

**Table 1:** Gender and age demographics of the COVID-19-naïve group (CNG) of vaccinees who developed breakthrough infections (BTIs) and the previously COVID-19-exposed group (CEG) of vaccinees who developed re-infections (RIs).

	COVID-19-Naïve (CNG)	COVID-19-Exposed (CEG)	CNG Breakthrough Infections (BTIs)	CEG Reinfections (RIs)
Overall (N = 205)	111 (54)	94 (46)	49 (44)	25 (27)
Gender				
Males (N = 56)	33 (59)	23 (41)	21 (64)	11 (48)
Females (N = 149)	78 (52)	71 (48)	28 (36)	14 (20)
*p* value	0.4		**0.007**	**0.008**
Age (years)				
20–44 (N = 140)	75 (54)	65 (46)	33 (44)	13 (20)
45–60 (N = 51)	28 (55)	23 (45)	12 (43)	9 (39)
≥61 (N = 14)	8 (57)	6 (43)	4 (50)	3 (50)
*p* value	1.0		0.9	0.08

*p* values were calculated by chi-squared tests; numbers in parentheses denote percentages; percentages of CNG and CEG columns were calculated over the corresponding N values given in the first column; percentages of BTIs were calculated over the numbers in the CNG column; percentages of RIs were calculated over the numbers in the CEG column.

**Table 2:** Median antibody levels and mean cytokine levels in sera from COVID-19 vaccinees.

Time Points	CNG (N)	CEG (N)	BTI (N)	RI (N)
**Median anti-SARS-CoV-2 spike Ig antibodies (U/mL)**				
1d-1m	84 (111)	10,152 (94)	ND	ND
2d-1m	856 (95)	6986 (83)	ND	ND
2d-3m	341 (48)	5372 (56)	4258 (45)	7188 (25)
2d-6m	153 (42)	3056 (50)	2386 (46)	11,096 (25)
**Mean IL-10 (pg/mL)**				
1d-1m	11 (24)	20 (36)	ND	ND
2d-1m	24 (24)	36 (36)	ND	ND
**Mean IL-17 (pg/mL)**				
1d-1m	2.24 (100)	4.30 (90)	ND	ND
2d-1m	2.75 (83)	5.42 (76)	ND	ND
**Mean IL-21 (pg/mL)**				
1d-1m	39 (100)	60 (90)	ND	ND
2d-1m	46 (83)	53 (76)	ND	ND
**3–6 months after second vaccine dose**				
Mean IL-7 (pg/mL)	10 (47)	12 (54)	18 (39)	14 (22)
Mean IL-15 (pg/mL)	21 (47)	19 (54)	23 (39)	12 (22)

N—number of samples tested; ND—not done; U—units; pg—picogram; mL—millilitre; d—vaccine dose; m—month.

## Data Availability

The data presented in this study are available in this article.
